# Impact of barcode medication administration on patient safety in UK hospital settings: protocol for a mixed-methods realist evaluation

**DOI:** 10.1136/bmjopen-2025-109619

**Published:** 2025-11-12

**Authors:** Aseel Mahmoud, Shahd Abdelaziz, Mairead McErlean, Yogini Jani, Mandy Slatter, Angelica Villena, James Bird, Kate Grailey, Alex Taylor, Bryony Dean Franklin

**Affiliations:** 1 Pharmacy, Imperial College Healthcare NHS Trust, London, UK; 2 Medical School, University of Exeter Faculty of Health and Life Sciences, Exeter, UK; 3 University College London Hospitals NHS Foundation Trust, London, UK; 4 Department of Practice and Policy, UCL School of Pharmacy, London, UK; 5 Royal United Hospital Bath NHS Trust, Bath, UK; 6 Imperial College Healthcare NHS Trust, London, UK; 7 Chelsea and Westminster Hospital NHS Foundation Trust, London, UK; 8 Surgery and Cancer, Imperial College London Faculty of Medicine, London, UK; 9 Patient and Public Involvement Partner, London, UK

**Keywords:** Safety, Delivery of Health Care, Integrated, Health policy, Health & safety, Digital Technology

## Abstract

**Abstract:**

**Introduction:**

Barcode medication administration (BCMA) systems are increasingly being implemented in hospital settings, with the aim of decreasing medication administration errors. However, the majority of the literature demonstrating the value of BCMA in supporting patient safety is from the USA. Furthermore, little is known about the underlying mechanisms that support its use. This study aims to explore the impact of BCMA on patient safety including medication admisntration errors and nursing time spent providing direct patient care, in terms of what works, for whom, under what circumstances, and how.

**Methods and analysis:**

We will use a mixed-methods realist evaluation. The study will be conducted in four phases, at two London NHS teaching trusts and one South West Region NHS Trust using different electronic health record systems. Phase 1 will involve documentary analysis and a narrative review to develop an initial programme theory for how BCMA is expected to work. Phase 2 will use interviews with key informants to refine this programme theory. The programme theory will then be tested in phase 3 using mixed methods: (1) observation of nurses’ medication administration; (2) analysis of alert data from the BCMA systems to understand the alerts’ clinical significance and utility and (3) interviews with nurses and hospital inpatients to explore their views. These data will be triangulated to refine and finalise the programme theory in phase 4, together with recommendations for practice.

**Ethics and dissemination:**

The Study Coordination Centre has obtained approval (24/SC/0326) from the Oxford B NHS Research Ethics Committee and the Health Research Authority. The study’s findings will be presented at scientific meetings and published in peer-reviewed journals. Additionally, summaries of the findings will be produced, targeted at relevant groups such as healthcare professionals, policy-makers and study participants.

**Strengths and limitations of this study**

- A strength of our study is that it will be conducted in three hospital trusts using two different electronic health record systems, providing broader generalisability.
- A further strength is use of alert data in combination with observed data, to ascertain whether alerts were in response to a potential error or spurious.
- A limitation in relation to our interviews with hospital staff is the risk of social desirability bias; our interviewers will adopt an open and non-judgemental approach to reduce this risk.
- Another limitation is the potential for observer bias to influence staff behaviour during the observations.

## Introduction

 Globally, medication errors are the leading cause of avoidable harms in healthcare.^1^ The global cost of medication errors is estimated to be US$42 billion annually.^2^ In England alone, medication errors are estimated to contribute to 1700 deaths, cause substantial morbidity and cost the NHS over £98 million per year.^3^ Errors most commonly occur at the stage of medication administration, with an estimated 37 million medication administration errors (MAEs) each year in English hospitals, of which 2.8 million (7.5%) are moderate or severe in terms of patient harm.^3^

Barcode medication administration (BCMA) technology is often advocated as a solution for reducing MAEs in the inpatient setting. This technology uses barcodes on patient wristbands and medication packaging to support adherence to the ‘five rights’ of medication administration (right patient, drug, dose, route and time) by alerting nurses to any mismatch between the medication ordered and the patient and medication scanned.

Four systematic reviews^4^,^7^ have explored the impact of BCMA on MAEs in the inpatient setting. These suggest that BCMA may reduce the incidence of MAEs such as wrong dose, medication, patient or route, but has little effect on wrong time errors. However, there is considerable variation in the results of included studies, suggesting that the impact may depend on how systems are implemented and used. Another further review examined human factor-related enablers and barriers to BCMA use and highlighted that a collaborative approach between system developers and end-users (usually nurses) is likely to be needed to achieve the intended safety outcome.^8^ Importantly, suboptimal compliance with BCMA scanning and the use of workarounds^9 10^ can introduce new risks to patient safety^8 11 12^ and may affect both implementation and sustained use of BCMA.^13^ Such studies highlight the need to identify in what circumstances BCMA is used as intended, and to better understand how BCMA can reduce MAEs and support patient safety.

Additionally, most existing research on BCMA use and its impact on patient safety originates from the USA.^4^,^7^ Differences in medicines management systems and practices between the USA and UK, as well as other countries, limit generalisability.^14^ For example, most medications in the USA are supplied in unit dose packaging and dispensed to individual patients, and/or are available only from ward-based automated dispensing cabinets.^14^ In the UK and many other countries, most medication is supplied as stock in original packs stored in ward medication rooms, with other medication dispensed to individual patients as multiple dose boxes or bottles as needed.^14^ Another difference is in the use of patients’ own medicines brought in from home, which is more common in countries outside of the USA.^14^ These supplies may be from different manufacturers (and thus have different barcodes) to the medication stocked in the hospital. As a result of these differences in medicines management practices, there may also be differences in the roles of nursing and pharmacy staff across different countries.^14^ Thus, findings relating to BCMA cannot necessarily be extrapolated from one specific country to other settings.

BCMA is clearly a complex intervention, with the available literature suggesting that it ‘works’ in different ways in different circumstances.^4^,^7^ For example, BCMA may work better for certain medications or formulations than for others. Overall, there is a need to better understand what works (in terms of the use of BCMA and how it may affect patient safety), for whom (staff, organisation and patients) and how (mechanisms to enhance BCMA use and its effect on patient safety). This can be gained through a realist evaluation,^15 16^ which is a theory-driven approach that can be used to evaluate complex interventions comprising multiple elements. The focus of realism is on the mechanisms of explanation,^15^ to provide an explicit, in-depth understanding of what works, for whom, under what circumstances and how. The findings will provide evidence to support decisions about the implementation of BCMA and how it can best be used to support patient safety.

### Aim and objectives

The aim of this study is to explore the impact of BCMA on patient safety (the ‘BCMAPS study’) including MAEs and nursing time spent providing direct patient care, in terms of what works, for whom, under what circumstances, and how.

Our objectives are to:

1. Develop an initial programme theory for how BCMA is expected to work in practice.
2. Test the initial programme theory.
3. Refine the programme theory to establish what works, for whom, under what circumstances, and how, in relation to BCMA and its impact on patient safety including nursing time spent providing direct patient care.
4. To make recommendations for practice and policy in relation to how to implement and use BCMA.

## Methods and analysis

### Study design

The BCMAPS study will be a realist evaluation^17 18^ with a mixed-methods design. Qualitative data will be collected during observations and semistructured interviews; quantitative data will be collected during the same observations together with analysis of BCMA alerts. The realist approach will allow us to explore the impact of mechanisms and contextual elements on what leads nurses to use BCMA in clinical practice, and how its use affects patient safety, including nursing time spent on direct patient care. The original organisational choice to implement BCMA is not in scope for this study.

**Figure 1:**
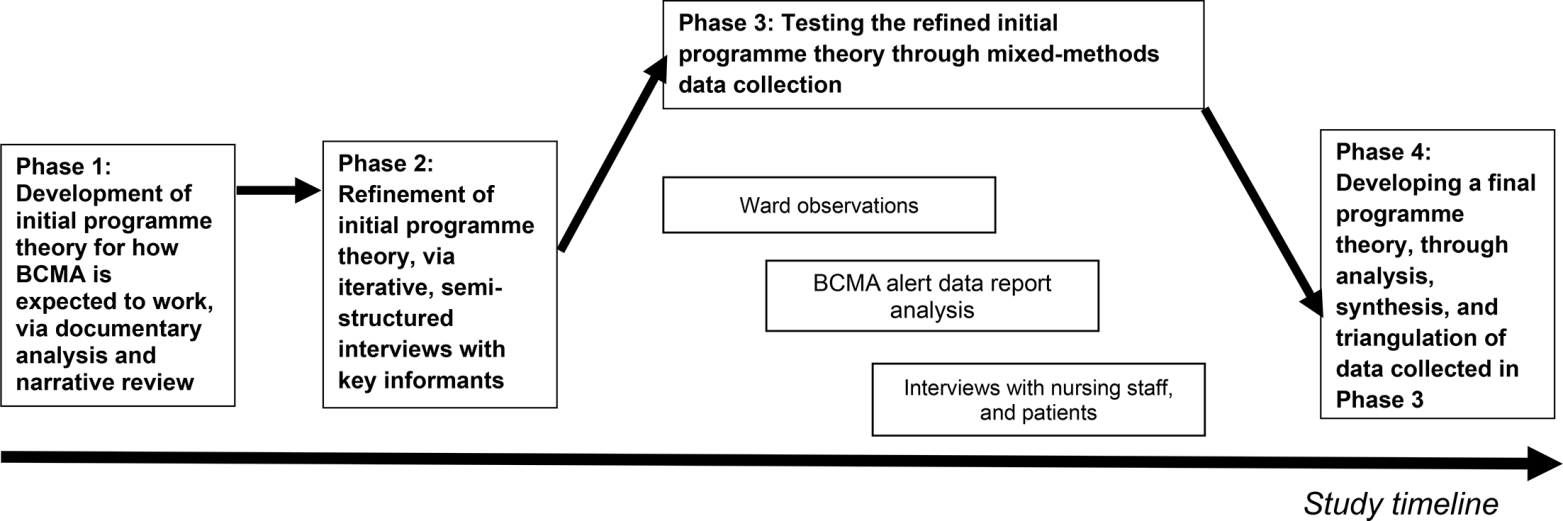
Overview of the study design. BCMA, barcode medication administration.

The study will be conducted in four phases (figure 1).

Phase 1 (November 2024 to February 2025) involves a narrative review to develop an initial programme theory; phase 2 (February to April 2025) uses interviews with key informants to refine the programme theory. Phase 3 then tests this programme theory (July to November 2025) using observation of nurses’ medication administration, analysis of alert data from the BCMA systems and interviews with nurses and hospital inpatients to explore their views. These data will be triangulated to refine and finalise the programme theory in phase 4 (January to March 2026), together with practice recommendations.

### Setting

The study will take place at three NHS teaching hospital trusts: two in London and one in the Southwest Region. Trust A has three main hospital sites and variable use of BCMA on inpatient wards based on local usage data. BCMA was implemented on some wards at the first site in 2019, followed by further roll-out at this and a second site in 2021–2022, and then at the third site during 2023. The trust uses Cerner as its electronic health record system. Trust B has four main inpatient sites. BCMA was implemented in all areas including outpatient and day case areas as part of a ‘big-bang’ electronic health record implementation in 2019. The use of BCMA is highly variable across the different inpatient sites and individual wards. This trust uses Epic as its electronic health record system. Trust C has one main inpatient site. BCMA was introduced in November 2017 as part of the ‘big bang’ electronic patient medicines administration system roll out in all adult areas other than the emergency department. Children’s inpatient areas were added in August 2021. Use is highly variable across different clinical areas. This trust uses Cerner Millennium as its electronic health record system.

### Phase 1: development of initial programme theory

The first phase comprises development of an initial programme theory for how BCMA is expected to work in practice, with a focus on its use by nurses and its impact on patient safety. This will be constructed as conjectured Context, Mechanism and Outcome configurations (‘CMOcs’); these are formulations of hypotheses about causal pathways within the paradigm of realist evaluation.^15^

The development of the programme theory will be informed by two main sources: relevant research literature surrounding BCMA use and implementation in hospitals, and documentary analysis (local, national and international guidelines, standard operating procedures and policy documents).

Documents identified from the literature search and relevant documents will be reviewed to develop the initial programme theory. The CMOcs framework will underpin an iterative process of reflection and adaptation to identify and articulate relationships among contexts, mechanisms and outcomes. Given that BCMA is a complex technology-based intervention, the CMOcs will also be mapped using the six domains of the non-adoption, abandonment, scale-up, spread, sustainability (NASSS) framework,^19^ which is an appropriate framework for evaluation technology-based complex interventions in practice.

### Phase 2: refinement of initial programme theory

The initial programme theory will then be presented to key informants in iterative, semistructured interviews to refine it, before testing in phase 3. Six key informants will be purposively recruited based on their knowledge/expertise/roles in the implementation and use of BCMA in the UK hospital inpatient setting, aiming for variation in roles and professions. A sample of six was chosen on the basis of the pool of experts in this field being relatively small. Following written informed consent, participants will be invited to discuss their reflections on the initial programme theory and to suggest areas of change or improvement, so that the initial programme theory can be refined. Semistructured topic guides (online supplemental additional file 1) will be used to facilitate the interviews. These will be iterative, with the associated prompts revised if needed after each interview. It is anticipated that interviews will be 20–45 min long. All participants will be invited to share their demographic information (age, gender, ethnicity and NHS Agenda for Change pay band if applicable) after their interview for the purpose of reporting characteristics of the study’s participants. Interviewees will be offered a £10 gift voucher for their participation.

### Phase 3: testing the refined initial programme theory

Testing the refined initial programme theory will involve a parallel convergent mixed methods approach using three data collection techniques: ethnographic ward observations (quantitative and qualitative), analysis of routine BCMA alert log data (quantitative) and interviews with nurses and patients (qualitative). All data collection will happen broadly in parallel (figure 1).

#### Ethnographic ward observations

Observations will take place on three adult, inpatient wards at each trust: one ‘high’ BCMA-use ward, one ‘medium’ use ward and one ‘low’ use ward. We will seek guidance from the trusts’ Chief Nursing Information Officers as to what constitutes ‘high’, ‘medium’ and ‘low’ use wards at the time of ward recruitment, seeking to use a consistent approach across the three trusts.

We aim to observe 400 doses of medication being prepared and administered on each of the three wards in each trust, to give 1200 in total from each study trust, in line with previous observation-based studies in this field.^10 20^ A sample of 1200 doses would allow a 5.6% MAE rate^21^ to be reported with a CI of 4.3% to 6.9% in each hospital. Based on previous studies,^20 22^ we anticipate being able to observe about 25 doses at each 8:00 round, 10 at each midday round and 10 at each 17:00 round. We will, therefore, aim to observe around 27 medication administration rounds on each ward, to include similar numbers of 8:00, midday and 17:00 medication administration rounds, and different nurses of different grades.

Prior to the time of a scheduled medication administration round, the researcher will provide nursing staff on the ward with a participant information sheet (PIS), inform them of the study and ask for a volunteer to be observed.

The researcher will accompany the nurse on the medication round to observe medication administration practices. Both quantitative and qualitative data will be collected (online supplemental additional file 2). Quantitative data (table 1) will include brief details of the doses administered (or omitted), whether medications have a barcode, whether they are a type of medication deemed to be scannable as per local policy, whether they were scanned, whether an alert is generated, and details of any potential MAEs prevented. Qualitative ethnographic observational data relating to the use of BCMA, how it fits nursing workflow, and any workarounds or challenges experienced will be recorded as free text. All data will be recorded during the observations using a paper data collection form; our previous work^20 22^ confirms this to be feasible.

**Table 1:** Quantitative variables to be measured during ward observations

Category	Variable(s)
Medication round	Ward, time of round, duration of round, number of patients to whom medication was administered during that round.
Doses	Number of doses administered, number of scannable doses, number of doses administered using BCMA, dosage forms, number of doses associated with ‘workarounds’.
Alerts	Number of alerts/types of alert and how nursing staff responded to these
Potential medication administration errors	Number and types of any potential medication administration errors and which of these were prevented by BCMA*
The time nurses spent on medication administration	Mean time nurses spent on medication administration per round, per patient and per dose.

BCMA, barcode medication administration.

If the researcher identifies a potential MAE, and/or no BCMA alert appears, or if the researcher is concerned that a nurse has inappropriately overridden an alert, the researcher will tactfully intervene to prevent the error from reaching the patient if the error is deemed to have potential for patient harm as outlined in online supplemental additional file 3.

The data collection method and data collection form (online supplemental additional file 2) will be piloted on a small number of medication administration rounds to test their usability prior to starting the main period of data collection. The six observers (two in trust A, two in trust B and three in trust C) will undergo training together, followed by discussion of coding of observations to ensure a consistent approach in data collection and analysis.

#### Analysis of routine BCMA alert log data

A member of trust clinical staff will request the routinely available alert data generated for each observed medication administration round. These reports will be used to match the alerts recorded with what happened in practice, based on the time and date, to identify which alerts were in response to a potential error and which were spurious. This will be done by a panel of researchers and clinical staff from the hospital trusts concerned who will be provided with fully anonymous scenarios outlining the medication that was due to be given and details of the alert. Data will be presented using descriptive statistics.

#### Interviews with nursing staff and patients

We will conduct up to 10 interviews with nursing staff and 10 with patients in each trust, to give 60 interviews in total. We anticipate that this number of interview participants will provide us with theoretical saturation and support review and refinement of the initial programme theory. Inclusion criteria for nursing staff will include nursing staff who work on adult inpatient wards providing direct patient care, including bank staff, or managerial staff involved with the use of BCMA. Inclusion criteria for patients will include being aged 18 years or older and able to provide informed, written consent.

Nursing staff will be recruited through volunteer sampling, where study information will be disseminated via trust communication networks; convenience sampling, where the research team will request the support of nursing and pharmacy colleagues to identify suitable nursing staff who may be interested in taking part; and purposive sampling, where more senior nursing staff such as trust BCMA champions, or those who have a managerial role that requires them to manage staff who use BCMA, will be contacted directly and invited to participate.

Patients will be recruited by convenience sampling. A member of the direct care team will make the first approach to potential participants. If a patient expresses an interest in finding out more, a member of the research team will then approach them to inform them of the study and invite them to take part. Patients will be given as much time as they need to read the PIS and decide whether or not to take part. Patients who consent to taking part will be given the option to be interviewed with an informal carer/relative/friend present if they prefer. Translation will be arranged for patients who wish to be interviewed in a language that is not spoken by the researcher.

All participants will be invited to discuss their perceptions and beliefs surrounding BCMA and its impact on patient safety and nursing time spent on direct patient care. Separate semistructured topic guides (onlinesupplemental additional files 4  5) will be used to facilitate the interviews with nursing staff and patients. The topic guides will be piloted with 2–3 participants prior to first use and will be iterative, building on earlier interviews and the developing programme theory.

It is anticipated that interviews will be 20–45 min long. If participants consent, interviews will be audio recorded and transcribed verbatim, with additional handwritten notes also taken. If participants do not consent to being recorded, detailed handwritten notes will be taken during interview discussions. Interview transcripts and handwritten notes will be pseudonymised and assigned arbitrary participant codes. Interviewees will be offered a £10 gift voucher for their participation.

### Data analysis

Qualitative data from interviews and observations will be analysed by AM, MM, BF and YJ using a combination of deductive and inductive analysis and will be conducted iteratively.

We define patient safety as ‘the avoidance of unintended or unexpected harm to people during the provision of healthcare’.^23^ We conceptualise healthcare as a complex sociotechnical system in which errors can occur. The Systems Engineering Initiative for Patient Safety (SEIPS) is an appropriate framework for understanding outcomes within complex sociotechnical systems. It allows exploration of the range of influences on how work is done in practice and has been widely used to study patient safety in different contexts. Therefore, deductive analysis will be based on the initial programme theory and may also draw on the SEIPS 2.0 model^24^ and NASSS framework. Inductive analysis will be based on reflexive thematic analysis to capture important themes outside of the deductive analysis.^25^

Quantitative data will be presented descriptively, describing the proportions of doses that were scannable, scanned, triggered alerts, associated with workarounds and any MAEs identified. Differences between wards and times of day will be examined in an exploratory analysis using appropriate univariate statistics (eg, χ² tests for categorical data). Associations between workarounds and any MAEs will be investigated by calculating and comparing ORs for MAEs. Comparison with routine BCMA alert log data will allow calculation of sensitivity and specificity of these data for identifying MAEs averted. Clinical significance of any MAEs averted will be assessed using the method of Dean and Barber.^26^

### Consent

All participants in phases 2 and 3 will be provided with a PIS and asked to give informed written consent.

### Demographic data collection

All participants (key informants, nursing staff and patients) in phases 2 and 3 will be invited to share their demographic information (age, gender, ethnicity, NHS Agenda for Change pay band where applicable) after the interview or observation for the purpose of reporting on the characteristics of the study’s participants.

### Phase 4: developing a final programme theory

The final stage will involve development of the final programme theory, based on the findings from phase 3. We will refine the initial programme theory through analysis and interpretation of existing data to establish how in different contexts (‘C’), various mechanisms (‘M’) are triggered to generate outcomes or results (‘O’). In line with the principles of realist evaluation, these will be described using CMOcs, drawing on principles of retroductive analysis, which refers to the identification of causal factors that lie behind identified patterns.^27^ We will also look for examples where anticipated outcomes are not achieved, identifying any ‘blocking mechanisms’.^28^ The final programme theory will be used to draw recommendations for practice on implementation of BCMA.

### Patient and public involvement

We presented an outline of this study to the NIHR North West London Patient Safety Research Collaboration’s Research Partners Group to explore the thoughts and reflections of patients and the public on the study’s importance and how we can most meaningfully involve patient and public involvement (PPI) partners. Participants felt that the work was important and advised that it would be beneficial to include PPI partners with experience of being hospital inpatients, ideally both with and without experience of BCMA technology.

We will, therefore, work with at least two PPI partners, who will be involved in shaping the study, developing and reviewing the study documents and interview guides (particularly those that are patient-facing), data analysis and synthesis, final report development and study dissemination.

PPI partners will be paid in accordance with the National Institue for Health and Care Research (NIHR) INVOLVE guidelines for their involvement and contributions to the study, and the impact of their involvement will be recorded throughout the study.^29^

### Reporting

The study will be reported using the Realist And Meta-narrative Evidence Syntheses: Evolving Standards (RAMESES) II reporting standards for realist evaluations^30^ and the Guidance for Reporting Involvement of Patients and the Public (GRIPP2) guidelines for reporting on PPI.^31^

## Ethics and dissemination

### Ethics approval and consent to participate

The Study Coordination Centre has obtained approval (24/SC/0326) from the Oxford B National Health Services (NHS) Research Ethics Committee and the Health Research Authority. The study will be conducted in accordance with the recommendations for physicians involved in research on human subjects adopted by the 18th World Medical Assembly, Helsinki 1964 and later revisions.

Consent to enter the study will be sought from each participant only after a full explanation has been given, an information leaflet offered and time allowed for consideration. Signed participant consent will be obtained.

### Dissemination

The findings of the study will be presented at scientific meetings and published in peer-reviewed journals. Additionally, summaries of the findings will be produced, targeted at relevant groups such as healthcare professionals, policy-makers and the participants who consent to having a summary of the findings sent to them at the end of the study.

### Consent for publication

All interview data will be fully anonymised before any analysis is undertaken, and all interview participants consented to the publication of anonymous quotes from interviews. Published quotes will be accompanied by arbitrary participant codes that will only describe the role of the participant (eg, doctor, key informant, nurse, patient), and will not be traceable back to individual participants.

### Availability of data and materials

The fully anonymised datasets will be available from the corresponding author on reasonable request for a period of 5 years.

## Supplementary material

10.1136/bmjopen-2025-109619 online supplemental file 1

10.1136/bmjopen-2025-109619 online supplemental file 2

10.1136/bmjopen-2025-109619 online supplemental file 3

10.1136/bmjopen-2025-109619 online supplemental file 4

10.1136/bmjopen-2025-109619 online supplemental file 5
