# Supplementary material for: Impact of barcode medication administration on patient safety in UK hospital settings: protocol for a mixed-methods realist evaluation
Source: BMJ Open. 2025 Nov 12;15(11):e109619. doi: 10.1136/bmjopen-2025-109619 (PMC12612751; doi:10.1136/bmjopen-2025-109619)
Supplement: online supplemental file 1 [file bmjopen-15-11-s001.docx]

**BCMAPS study**

**DRAFT KEY INFORMANT PARTICIPANT INTERVIEW TOPIC GUIDE**

The impact of **B**ar**C**ode **M**edication **A**dministration on **P**atient **S**afety (BCMAPS) in UK hospital settings: a mixed-methods realist evaluation

**IRAS ID:** **338756**

**REC reference: (24/SC/0326)**

**Instructions for the researcher**

**Confirm that the interviewee understands:**

- The purpose of the research
- What the interview will entail
- How confidentiality and anonymity will be assured
- That they can stop at any time without explanation

**And:**

- They have had the chance to ask questions
- They have given informed consent to take part in the study and to be interviewed
- Whether or not they consent to being audio recorded

**Interview questions**

1. What are your opinions of the use of BCMA scanners in inpatient hospital settings?

*Prompts*

1. *What are your thoughts on why they’re being implemented?*
   - ***Why do you think this?***
2. I’m wondering if there are situations where BCMA may be more helpful for nurses, and situations where it may be less helpful
3. What are your thoughts on this?

*Prompts*

- *What are your opinions on the types of wards or medication it will work well for, or for which types of nursing staff?*
- Are they any situations or types of wards where you think BCMA may not be helpful?
  - ***Why do you think this?***

1. What do you think needs to be put in place for BCMA to be most helpful?
   - ***Why do you think this?***

*Prompts*

- *What are your opinions on any changes or improvements to the BCMA system or the hospital ward environment that may enhance BCMA’s value in safe medication administration/support nurses to use it?*
  - ***Why do you think this?***

1. As BCMA is being introduced more and more in hospitals, I’m wondering if there are any organisational characteristics or processes that would support the use of BCMA in an organisation’s inpatient wards?

What are your thoughts on this?

*Prompts*

- - *What do you think makes BCMA use successful in a hospital organisation?*
    - ***Why do you think this?***

1. What are your opinions on the roles of different groups of hospital staff in supporting the use of BCMA?

*Prompts*

- - *Which groups of staff do you think should be involved in supporting the use of BCMA, and how?*
    - ***Why do you think this?***

1. From past studies, we know that BCMA may help nurses give medications out to patients safely, but nurses also can experience problems when they use BCMA.

What do you think of this?

1. I’m wondering if using BCMA will have an effect on nurses interacting with patients. What do you think about this?

*Prompts*

- *How do you think it will affect how nurses give people their medication?*
- *What do you think about nurses using BCMA affecting the time it takes them to administer medication to patients?*
  - ***Why do you think this?***

1. What are your opinions or knowledge of the evidence surrounding BCMA supporting patient safety?
   - ***Why do you think this?***
2. *What are your opinions, if any, on the training and support that should be provided to nurses to support their use of BCMA?*
   - ***Why do you think this?***
3. That was the last of the interview questions. Do you have anything else that you would like to share that I have not asked you about, about BCMA, and where you think it works/may work well, or does not/may not work well?

**Instructions for the researcher:**

- Thank participant for their time, ask if they have any questions.
- Summary, wrap up, next steps if participant requested to receive a summary of the results and/or a gift voucher.
