# Supplementary material for: Impact of barcode medication administration on patient safety in UK hospital settings: protocol for a mixed-methods realist evaluation
Source: BMJ Open. 2025 Nov 12;15(11):e109619. doi: 10.1136/bmjopen-2025-109619 (PMC12612751; doi:10.1136/bmjopen-2025-109619)

*Decision aid for the study’s researchers on when and how to intervene and/or report potential medication administration errors*


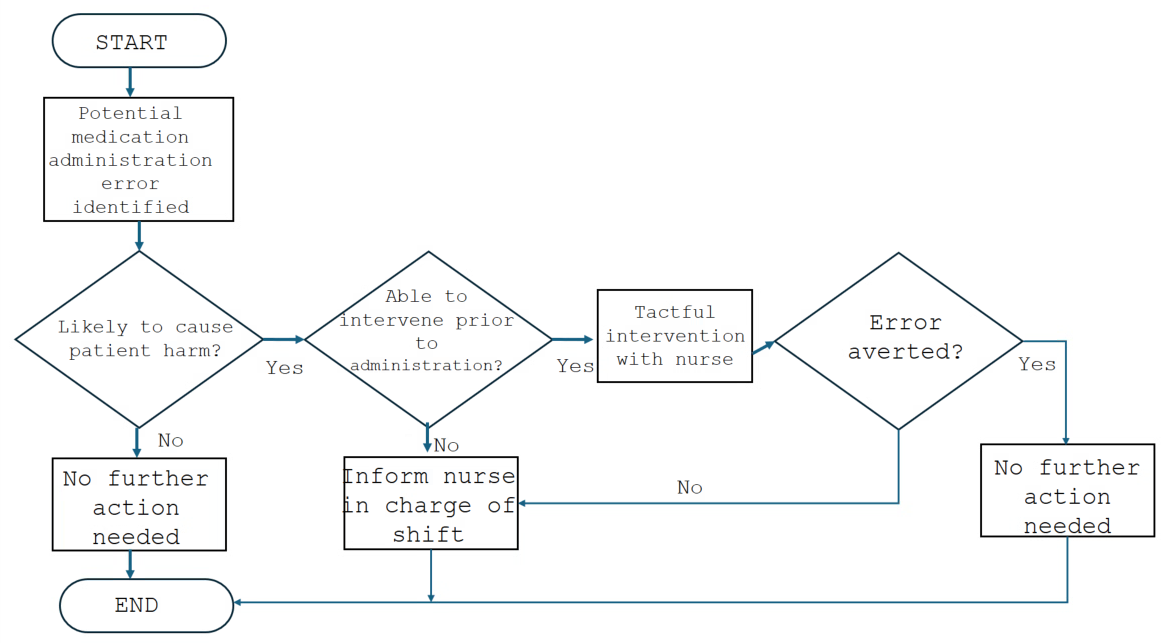

Supplement: online supplemental file 3 [file bmjopen-15-11-s003.docx]
