# Supplementary material for: Impact of barcode medication administration on patient safety in UK hospital settings: protocol for a mixed-methods realist evaluation
Source: BMJ Open. 2025 Nov 12;15(11):e109619. doi: 10.1136/bmjopen-2025-109619 (PMC12612751; doi:10.1136/bmjopen-2025-109619)
Supplement: online supplemental file 4 [file bmjopen-15-11-s004.docx]

**BCMAPS study**

**DRAFT PATIENT PARTICIPANT INTERVIEW TOPIC GUIDE**

The impact of **B**ar**C**ode **M**edication **A**dministration on **P**atient **S**afety (BCMAPS) in UK hospital settings: a mixed-methods realist evaluation

**IRAS ID:** **338756**

**REC reference: (24/SC/0326)**

**Instructions for the researcher**

**Confirm that the interviewee understands:**

- The purpose of the research
- What the interview will entail
- How confidentiality and anonymity will be assured
- That they can stop at any time without explanation

**And:**

- They have had the chance to ask questions
- They have given informed consent to take part in the study and to be interviewed
- Whether or not they consent to being audio recorded

**Interview questions**

1. Nurses sometimes use barcode medication administration, or BCMA, when they give patients their medication. They use handheld scanners to scan the barcode on patients’ wristbands and on the medication boxes.
   1. Have you seen the nurses use these, on this, or any past admissions to hospital? (***possibly point to a BCMA scanner if one is available).***

***If patient answers ‘no’ to above question or cannot recall seeing BCMA, reiterate explanation of what BCMA is, if needed (handheld scanners to scan the barcode on patients’ wristbands and on the medication boxes), and reframe upcoming questions for patients to ‘imagine’ what BCMA/their experiences/their opinions may be like.***

- 1. Do you have any opinions about their use?

*Prompts*

- *Why do you think they are being used?*
  - ***Why do you think this?***

1. What have been your experiences when nurses have used BCMA to scan your wristband or your medication?

*Prompts*

- *What are your experiences of the nurses interacting with you when they’ve used BCMA?* *(On this, or past admissions)*
  - ***What do you think contributed to this?***

1. One of the reasons BCMA is being introduced in hospitals is because it’s believed that it helps nurses give patients their medications safely.

What do you think about this?

*Prompts*

- *How do you think it affects how nurses give people their medication?*
  - ***Why do you think this?***

1. From past studies, we know that BCMA may help nurses give medications out to patients safely, but nurses also can experience problems when they use BCMA.

Have you experienced or seen any problems that the nurses had with BCMA? (*On this, or past admissions)*

*Prompts*

- *What did you think of this?*
- *How do you think the problem affected the nurse?*
  - ***What do you think contributed to this?***

1. I’m wondering if there are situations where BCMA may be more helpful for nurses, and situations where it may be less helpful

What are your thoughts on this?

- - - ***Why do you think this?***

1. I am wondering if sometimes nurses have to spend time sorting out these problems.

What do you think about this?

1. Nurses do not always use BCMA, for various reasons, and sometimes they will give people their medication without using it.
2. What have been your experiences in the differences between nurses using and not using BCMA?

*Prompts*

- *How have you felt when they gave out your medication with and without BCMA? (On this, or past admissions)*
  - ***Why do you think this?***
  - ***What do you think contributed to this?***

1. I am thinking how nurses have interacted with you, what have been your experiences of the length of time nurses spend giving your medication with and without BCMA?

- ***What do you think contributed to this?***

1. What things do you think hospitals should do to make sure that nurses are supported to give patients their medication safely?
2. That was the last of the interview questions. Do you have anything else that you would like to share that I have not asked you about, about BCMA?

**Instructions for the researcher:**

- Thank participant for their time, ask if they have any questions.
- Summary, wrap up, next steps if participant requested to receive a summary of the results and/or a gift voucher.
