# Supplementary material for: Impact of barcode medication administration on patient safety in UK hospital settings: protocol for a mixed-methods realist evaluation
Source: BMJ Open. 2025 Nov 12;15(11):e109619. doi: 10.1136/bmjopen-2025-109619 (PMC12612751; doi:10.1136/bmjopen-2025-109619)
Supplement: online supplemental file 5 [file bmjopen-15-11-s005.docx]

**BCMAPS study**

**DRAFT NURSE PARTICIPANT INTERVIEW TOPIC GUIDE**

The impact of **B**ar**C**ode **M**edication **A**dministration on **P**atient **S**afety (BCMAPS) in UK hospital settings: a mixed-methods realist evaluation

**IRAS ID:** **338756**

**REC reference: (24/SC/0326)**

**Instructions for the researcher**

**Confirm that the interviewee understands:**

- The purpose of the research
- What the interview will entail
- How confidentiality and anonymity will be assured
- That they can stop at any time without explanation

**And:**

- They have had the chance to ask questions
- They have given informed consent to take part in the study and to be interviewed
- Whether or not they consent to being audio recorded

**Interview questions**

**Ward nurses:** What are your opinions and experiences of using BCMA scanners?

**Nurse managers:** What are your opinions and experiences, and/or the experiences of your teams, of using BCMA?

*Prompts*

1. *Why do you think they are being implemented?*
   - ***What do you think about this? / Why do you think this?***
2. I’m wondering if there are situations where BCMA may be more helpful for nurses, and situations where it may be less helpful
3. What are your thoughts on this?
   - ***Why do you think this?***
4. What do you think needs to be put in place for BCMA to be most helpful for nurses?

*Prompts*

- *What are your opinions on the types of wards or medication it will work well for, for which type of nursing staff?*
  - ***Why do you think this?***
- *What are your opinions on any changes or improvements to the BCMA system that may enhance its value in safe medication administration/support nurses to use it?*
  - ***Why do you think this?***

**Ward nurses:** What are your experiences of interacting with patients while using BCMA?

**Nurse managers:** What are your own experiences, or the experiences of your teams, of interacting with patients whilst using BCMA?

*Prompts*

- - ***What do you think contributes to this?***

1. One of the reasons BCMA is being introduced in hospitals is because it’s believed that it helps nurses give patients their medications safely.

What do you think about this?

*Prompts*

- *How do you think it will affect how nurses give people their medication?*
- *What do you think about nurses using BCMA affecting the time it takes for them to administer medication to patients?*
  - ***Why do you think this?***

1. From past studies, we know that BCMA may help nurses give medications out to patients safely, but nurses also can experience problems when they use BCMA.

**Ward nurses:** What are your experiences of problems when using BCMA?

**Nurse managers:** What are your own experiences, or the experiences of your teams, of problems when using BCMA?

*Prompts*

- *What did you think of this?*
- *How do you think the problem affected you/the nurses/safe administration of medication?*
  - ***What do you think contributed to this?***

1. I am wondering if sometimes nurses have to spend time sorting out these problems.

What do you think about this?

- - ***Why do you think this?***

1. Nurses do not always use BCMA, for various reasons - sometimes they might give people their medication without using it.
2. What have been your experiences in the differences between using and not using BCMA?

*Prompts*

- *How do you feel when you use BCMA compared to when not using BCMA?*
- *What do you feel works well when using BCMA, and when not using BCMA?*
  - ***What do you think contributes to this?***
  - ***Why do you think this?***

1. How do you feel about the way BCMA is being implemented at the trust, and how nurses are being encouraged to use it?

*Prompts*

- *What are your experiences of the training and support provided surrounding BCMA?*
  - ***What do you think contributes to this?***
  - ***Why do you think this?***

1. That was the last of the interview questions. Do you have anything else that you would like to share that I have not asked you about, about BCMA?

**Instructions for the researcher:**

- Thank participant for their time, ask if they have any questions.
- Summary, wrap up, next steps if participant requested to receive a summary of the results and/or a gift voucher.
