# Supplementary material for: Impact of barcode medication administration on patient safety in UK hospital settings: protocol for a mixed-methods realist evaluation
Source: BMJ Open. 2025 Nov 12;15(11):e109619. doi: 10.1136/bmjopen-2025-109619 (PMC12612751; doi:10.1136/bmjopen-2025-109619)
Supplement: Supplementary file 6 [file bmjopen-2025-109619.reviewer_comments.pdf]

PEER REVIEW HISTORY

BMJ Open publishes all reviews undertaken for accepted manuscripts. Reviewers are asked to complete a checklist review form and are provided with free text boxes to elaborate on their assessment. These free text comments are reproduced below.

ARTICLE DETAILS

Title (Provisional)

The impact of Barcode Medication Administration on Patient Safety in UK hospital settings: protocol for a mixed-methods realist evaluation

Authors

Mahmoud, Aseel; Abdelaziz, Shahd; Mcerlean, Mairead; Jani, Yogini; Slatter, Mandy; Villena, Angelica; Bird, James; Grailey, Kate; Taylor, Alex; Franklin, Bryony

---

VERSION 1 - REVIEW

**Reviewer**	**1**
**Name**	**Haugen, Arvid**
**Affiliation**	**Oslo Metropolitan University**
**Date**	**21-Sep-2025**
**COI**	**None**

Thank you for the opportunity to review this study protocol. This an interesting and complex study, and it is well presented. There may be a few points that could improve its methodological rigor.

In the Methods' section, reliability between observers is not described. To strengthen the study's reliability - a test of intra-rater reliability, using Kappa analysis between raters' observations would be helpful.

Impact on nursing time spent on direct patient care – how is this to be measured?

For the qualitative analysis a retroductive analysis approach will be used. Is this the same as an abductive approach? For the inductive analysis will there be a pragmatic content analysis or a thematic analysis? This could be more detailed.

The deductive analysis 'may also' draw on a patient safety framework (SEIPS or NASS). If these frameworks are to be used, an explanation on the authors' theoretical viewpoints on patient safety would be helpful – perhaps a definition on patient safety, in the method section.

The statistic section could be more detailed in this study protocol. Which variables (and measure levels) are to be analyzed in the univariate analysis by using Chi-squared test? Will

there be used any regression analyses, and which models will be applied? Especially, will there be any analysis on time spent on direct patient care (if that is a variable)? There is no sample size calculation provided for the quantitative data collection. Could you add this and include a reference on expected levels of changes (or associations) for the outcomes? This would have strengthened the quantitative part of the study.

There is a lack of references on page 4, lines 53-60.

VERSION 1 - AUTHOR RESPONSE

	Reviewer: 1<br>Dr. Arvid Haugen, Oslo Metropolitan University	Authors' response	Relevant changes in text
1	Thank you for the opportunity to review this study protocol. This is an interesting and complex study, and it is well presented. There may be a few points that could improve its methodological rigor.	Thank you for showing interest in our study. We have addressed your comments. Detailed response below.	NA
2	In the Methods' section, reliability between observers is not described. To strengthen the study's reliability - a test of intra-rater reliability, using Kappa analysis between raters' observations would be helpful.	Pilot work suggested that having two researchers observe the same nurse was not possible, due to lack of space in clinical areas. Instead, we supported reliability by providing training that all observers attended together, followed by discussion of coding of observations to ensure a consistent approach. We have now updated the paper to include this important point.	P10. L217-219
3	Impact on nursing time spent on direct patient care – how is this to be measured?	This will be explored during the qualitative interviews with nurses and patients. We have now clarified this in the methods section.	Methods<br>P11, L250-251
4	For the qualitative analysis a retroductive analysis approach will be used. Is this the same as an abductive approach? For the inductive analysis will there be a pragmatic content analysis or a thematic analysis? This could be more detailed.	The terms are sometimes used interchangeably. However, within realist analysis they are generally seen as different but complementary approaches. Specifically, realist analysis involves inductive, deductive (and sometimes abductive) approaches which then inform retroductive analysis to build and/or test the program theory. This is in line with how we describe our methods in the paper.	Methods<br>P12, L2624-263<br>L 271-272

		Reference: Realist analysis of qualitative data in health professions education research - PMC<br>Inductive analysis will be based on reflexive thematic analysis to capture important themes outside of the deductive analysis, as outlined in the paper.	
5	The deductive analysis 'may also' draw on a patient safety framework (SEIPS or NASS). If these frameworks are to be used, an explanation on the authors' theoretical viewpoints on patient safety would be helpful – perhaps a definition on patient safety, in the method section	We have clarified that "Patient safety is the avoidance of unintended or unexpected harm to people during the provision of health care".<br>Reference: NHS England » Patient safety<br>We conceptualise healthcare as a complex sociotechnical system in which errors can occur and this can impact patient safety. SEIPS, therefore is appropriate as a framework for understanding outcomes within complex socio-technical systems. BCMA is a complex intervention that utilises technology, therefore NASSS is an appropriate framework for the evaluation of the implementation of technology-based complex interventions in practice. We have now strengthened these justifications in the paper.	Methods<br>P12, L262-272
6	The statistic section could be more detailed in this study protocol. Which variables (and measure levels) are to be analysed in the univariate analysis by using Chi-squared test? Will there be used any regression analyses, and which models will be applied? Especially, will there be any analysis on time spent on direct patient care (if that is a variable)? This would have strengthened the quantitative part of the study.	We have updated the analysis section to address these important points.<br>We have now added Table 1, which includes all the variables that we will measure in the observations.<br>In the analysis section we have stated that all these will be analysed using descriptive statistics.<br>Additionally, we will compare some variables using Chi squares test. eg. Percentage scan rates between the three hospitals. . .	Methods<br>P10 Table 1<br>P, L 273-280
7	There is no sample size calculation provided for the quantitative data collection. Could you add this and include a reference on	Please see our response to the editor's comment 3 above.	Methods<br>P8-9, L187-189<br>P11, L230-231

	expected levels of changes (or associations) for the outcomes?		
8	There is a lack of references on page 4, lines 53-60.	We have updated the introduction and added appropriate references.	Introduction P4, L71-88
